# Predicting cardiovascular events with fluoropyrimidine chemotherapy using a standard cardiovascular risk calculator

**DOI:** 10.1002/ehf2.14879

**Published:** 2024-06-06

**Authors:** Aderonke Abiodun, Marianne Shawe‐Taylor, Sara Tyebally, Emmanouil Bagkeris, Omotomilola Bajomo, Jessica Artico, Sarah Slater, Zahra Raisi‐Estabragh, Nikolaos Diamantis, Charlotte Manisty

**Affiliations:** ^1^ Barts Heart Centre Barts Health NHS Trust London UK; ^2^ Institute of Cardiovascular Science University College London London UK; ^3^ National University Health System Singapore Singapore; ^4^ Great Ormond Street Institute of Child Health London UK; ^5^ Barts Cancer Centre Barts Health NHS Trust London UK; ^6^ William Harvey Research Institute Queen Mary University London London UK; ^7^ Department of Medical Oncology Royal Free London NHS Foundation Trust London UK

**Keywords:** 5‐FU, Capecitabine, Cardio oncology, Cardiotoxicity, Fluoropyrimidines, Risk prediction, QRISK

## Abstract

**Aims:**

Fluoropyrimidine chemotherapy is important for treatment of many solid tumours but is associated with cardiotoxicity. The relationship of fluoropyrimidine‐associated cardiotoxicity (FAC) with conventional cardiovascular (CV) risk factors is poorly understood, and standard cardiovascular risk scores are not validated in this context.

**Methods and results:**

Single‐centre retrospective study of patients treated with fluoropyrimidine chemotherapy using electronic health records for cardiovascular risk factors (and calculation of QRISK3 score), cancer treatment, and clinical outcomes. FAC was defined by cardiovascular events during or within 3 months of fluoropyrimidine treatment, and Cox regression was used to assess associations of CV risk and cancer treatment with FAC. One thousand eight hundred ninety‐eight patients were included (45% male; median age 64 years), with median follow up 24.5 (11.5–48.3 months); 52.7% of patients were at moderate or high baseline CV risk (QRISK3 score >10%) Cardiovascular events occurred in 3.1% (59/1898)—most commonly angina (64.4%, 38/59) and atrial fibrillation (13.6%, 8/59), with 39% events during cycle one of treatment. In univariable analysis, QRISK3 score >20% was significantly associated with incident FAC (HR 2.25, 95% CI 1.11–4.93, *P* = 0.03). On multivariable analysis, beta‐blocker use (HR 1.04, 95% CI 1.00–1.08, *P* = 0.04) and higher BMI (HR 2.33, 95% CI 1.04–5.19, *P* = 0.04) were independently associated with incident CV events. Thirty‐two of the 59 patients with FAC were subsequently rechallenged with fluoropyrimidine chemotherapy, with repeat CV events in 6% (2/32). Incident FAC did not affect overall survival (*P* = 0.50).

**Conclusions:**

High BMI and use of beta‐blockers are associated with risk of CV events during fluoropyrimidine chemotherapy. QRISK3 score may also play a role in identifying patients at high risk of CV events during fluoropyrimidine chemotherapy. Re‐challenge with further fluoropyrimidine chemotherapy can be considered in patients following CV events during prior treatment.

## Introduction

Fluoropyrimidines including 5‐fluorouracil (5‐FU) and capecitabine are the third most common cancer treatment class used worldwide.^1^ They form the basis of chemotherapy treatment for gastrointestinal malignancies, breast, pancreatic and head and neck cancers.^2^, ^3^, ^4^ For many patients who develop complications with 5‐FU, alternative cancer treatment options are very limited, particularly in colorectal cancer where 5‐FU is a backbone of first‐ and second‐line chemotherapy, and maintenance treatment.^5^

Cardiotoxicity is an important complication of fluoropyrimidine treatment and can lead to treatment interruption or cessation. Reported fluoropyrimidine associated cardiotoxicity (FAC) events include sudden death, angina, acute coronary syndromes, cardiac arrhythmias, and heart failure,^6^ which most commonly occur within the first cycle of treatment.^7^, ^8^ Reported incidence varies widely across studies, between 1.0% and 34.6%.^9^

Data regarding the relationship of FAC with conventional cardiovascular risk factors are conflicting; some studies showing a clear association^10^, ^11^ and others finding none.^7^, ^12^ Previous studies and clinical trials have excluded patients with pre‐existing cardiac diseases,^13^ and in some centres, clinicians withhold fluoropyrimidine chemotherapy from patients at high cardiovascular risk, although there are currently no validated risk prediction models for this approach.

A joint position statement from the International Cardio‐Oncology Society (ICOS) and the Cardio‐Oncology Study Group of the Heart Failure Association of the European Society of Cardiology has developed specific baseline cardiovascular risk stratification proformas for patients receiving a broad range of cancer therapies,^14^ but not for patients receiving fluoropyrimidines specifically—largely because of the absence of consistent evidence. Improved methods for identification of patients at highest risk would help minimize cardiotoxic events, reduce inappropriate withholding of first line fluoropyrimidine chemotherapy, and help target administration of cardioprotective medications.

This study presents a retrospective electronic health record (EHR) review of consecutive patients receiving fluoropyrimidines for any indication between 2014 and 2020 in a single tertiary centre. We characterized incident cardiovascular events occurring within 3 months of treatment exposure and examined association of baseline vascular risk factors and pre‐existing cardiovascular disease (CVD) with risk of events. Furthermore, we evaluated the utility of QRISK3,^19^ an established CVD risk score, for prediction of FAC. Finally, we assessed overall survival rates in patients with and without CV events during fluoropyrimidine treatment.

## Methods

### Setting and study population

We performed a retrospective observational analysis of all consecutive patients treated with a 5‐FU or capecitabine based chemotherapy regimen between January 2014 and October 2020 at Barts Cancer Centre, Barts Health NHS Trust, a tertiary level Cancer centre serving a socioeconomically and ethnically diverse population of 1.5 million in East London, UK. Institutional approval for the work was provided (Barts Health Audit code 10657).

### Defining treatment exposure and cancer characteristics

Adults (>18 years old) receiving 5‐FU or capecitabine were identified using the electronic chemotherapy prescription programme (ARIA Oncology, Varian, Palo Alto, California), which includes records of all chemotherapy prescriptions and their dates of administration. Patients receiving treatment in both the neoadjuvant, adjuvant, and metastatic setting with at least 6 months of follow‐up data were included. The outpatient and pharmacy EHRs were manually reviewed to collect information regarding cancer type, stage, patient performance status (PS), anticancer regimen, treatment setting (neoadjuvant, adjuvant, or palliative), and total number of treatment cycles received.

### Ascertainment of outcomes

The primary outcome was combined CV events associated with fluoropyrimidines (FAC). Aligned with previous studies,^1^ we defined FAC as composite of first occurrence of any of the following diagnoses at any time during receipt of fluoropyrimidine chemotherapy and up to 3 months after last chemotherapy administration:

- new onset angina (with or without ECG changes)
- acute coronary syndrome ‐ ACS: ST elevation myocardial infarction (STEMI), non‐ST‐elevation myocardial infarction (NSTEMI), and unstable angina
- new onset arrhythmia (atrial fibrillation (AF), other supraventricular tachycardia (SVT), ventricular tachycardia (VT), bradyarrhythmia requiring treatment)
- new onset of symptoms or signs of heart failure with or without left ventricular systolic dysfunction on cardiac imaging
- cardiac arrest where an alternative cause was deemed to be unlikely
- cardiovascular death

In individuals who developed FAC, we considered rates of chemotherapy discontinuation, chemotherapy re‐challenge, and overall survival (vs those without FAC) as secondary study outcomes.

Primary and secondary outcomes were determined by manual review of patients' EHRs to identify documented clinical diagnosis of the conditions of interest and the date of first occurrence. Diagnostic labels recorded in hospital inpatient and outpatient settings, and in linked primary care records were identified. We further scanned patient records for relevant International Classification of Diseases codes (*Table* *S1*). Endpoint adjudication was performed by two independent cardiologists (A. A. and C. H. M.), including review of cardiac investigations.

### Ascertainment of covariates

Manual chart review of EHRs allowed ascertainment of baseline (pre‐treatment) demographics, medical history, and follow up information.

The following information was collected based on known associations with cardiovascular risk: age, sex, ethnicity, body mass index (BMI), smoking status, diabetes, hypertension, hypercholesterolaemia, chronic kidney disease (as defined by eGFR <60 or stage III–V), peripheral vascular disease, and pre‐existing CVDs (ischaemic heart disease, cardiomyopathy, arrhythmias, valvular heart disease, heart failure, and stroke). Arrhythmias were defined as record of AF, atrial flutter, other SVT, VT, or the presence of a permanent pacemaker. Age was taken at the time of first chemotherapy administration. Sex and ethnicity were taken from EHRs. BMI and body surface area (BSA) were calculated from height and weight measured for chemotherapy prescription. Smoking was as per self‐report. Morbidity data and their date of occurrence were ascertained through secondary and primary care records. Ischaemic heart disease was defined as a history of coronary artery bypass grafting (CABG), previous percutaneous coronary intervention (PCI), or a medically managed myocardial infarction (MI). Baseline usage of the following cardiac medications was noted: ACE inhibitors, beta‐blockers, calcium channel blockers (CCBs), antiplatelets, anticoagulants, nitrates, and statins. Dihydropyrimidine dehydrogenase (DPD) mutation assessment results were collected where available.

Cardiac risk factors were manually inputted into the QRISK3^15^ online calculator to derive an estimate of 10‐year absolute risk of developing cardiovascular disease. This score has been developed and extensively validated in the general population. QRISK3 scores were divided into ‘low’ (<10%), ‘moderate’ (10.01–19.9%), and ‘high’ (>20%) risk categories as per published guidelines.^14^

### Statistical analysis

Statistical analysis was performed using the GraphPad Prism software (Version 9.4.0, https://www.graphpad.com/). Baseline patient characteristics were expressed as count and percentage for categorical variables, and either median with interquartile range (IQR) or mean with standard deviation for continuous variables based on data distribution.

Univariable Cox proportional hazard regression was used to estimate association of each covariate with incident FAC. Time zero was the date of first chemotherapy administration. All covariates were ascertained at baseline (pre‐chemotherapy). The FAC outcome was a composite outcome of incident cardiovascular diagnoses as defined previously, occurring during fluoropyrimidine chemotherapy and up until 3 months after last treatment exposure. Patients were censored when they experienced the outcome of interest, at 3‐months post chemotherapy completion, or if they were lost to follow up. Covariates found to be statistically significant (*P* < 0.05) on univariable analyses were included in multivariable analysis. Ethnicity was not included in multivariable models due to high missingness. We considered the association of QRISK3 score as a covariate, to evaluate its value as a potential predictor of FAC outcomes. QRISK3 was not included in multivariable analysis given significant co‐linearity with other covariates which form part of the score. Results are reported as hazard ratio (HR), 95% confidence interval (CI), and related *P*‐values.

Overall survival in those with and without FAC was analysed using univariable Cox regression, over a median follow up period of 2 (1.0–4.0) years. Patients were censored on death or at the end of the available follow up period.

## Results

### Baseline patient characteristics

A total of 1934 patients received fluoropyrimidine‐based chemotherapy during the study period. We excluded 36 individuals who were lost to follow up. The remaining 1898 individuals were included in the analysis, with a median age of 64 (IQR 54–72) years old and comprising 1045 (55.1%) women (*Table* *1*). The cohort included 42.9% Caucasian, 12.7% Black, 10.6% South Asian, 4.5% other Asian, and 12.2% ‘other’ ethnicities. Most patients (*n* = 975, 51.3%) had baseline PS of zero or one (*n* = 825, 43.5%).

**Table 1:** Baseline demographics and cancer type and treatment characteristics for patients with and without fluoropyrimidine induced cardiotoxicity

	Total patients (*n* = 1898)	Patients with FAC (*n* = 59)	Patients without FAC (*n* = 1839)
Age in years			
Median (IQR)	64 (54–72)	58 (46–67)	60 (50–68)
Male, *n* (%)	853 (44.9)	34 (57.6)	819 (44.5)
Ethnicity, *n* (%)			
White	815 (42.9)	31 (52.5)	784 (42.6)
Black	241(12.7)	4 (6.8)	237 (12.9)
South Asian	201 (10.6)	8 (13.6)	193 (10.5)
Other Asian	86 (4.5)	0 (0.0)	86 (4.7)
Other	232 (12.2)	14 (23.7)	218 (11.9)
Missing	323 (17.0)	2 (3.4)	321 (17.5)
Performance status, *n* (%)			
0	975 (51.3)	28 (47.5)	947 (51.5)
1	825 (43.5)	28 (47.5)	797 (43.3)
2	91 (0.5)	3 (5.1)	88 (4.8)
3	7 (0.4)	0 (0.0)	7 (0.4)
4	0 (0.0)	0 (0.0)	0 (0.0)
Primary tumour site, *n* (%)			
Colorectal	922 (48.6)	46 (78.0)	876 (47.6)
Breast	414 (21.8)	2 (3.4)	412 (22.4)
Oesophageal	150 (7.9)	3 (5.1)	147 (8.0)
Pancreas	132 (7.0)	2 (3.4)	130 (7.1)
Stomach	125 (6.6)	3 (5.1)	122 (66.3)
Head and neck	54 (2.8)	0 (0.0)	54 (2.9)
Hepatobiliary	43 (2.3)	2 (3.4)	41 (22.2)
Bladder	21(1.1)	0 (0.0)	21 (1.1)
Cancer of unknown primary	19 (1.0)	0 (0.0)	19 (1.0)
Other	18 (0.9)	1 (1.7)	17 (0.9)
Chemotherapy, median (IQR)			
Number of cycles received	6 (3–12)	6 (3–11)	6 (3–10)
Length of treatment (months)	4.2 (2.0–6.9)	3.8 (1.9–9.5)	3.9 (1.9–6.4)
Treatment intent, *n* (%)			
Curative	853 (44.9)	31 (52.5)	822 (44.7)
Palliative	1045 (55.1)	28 (47.5)	1017 (55.3)
Capecitabine, *n* (%)			
Single agent	196 (10.3)	8 (13.6)	188 (10.2)
Combination therapy^a^	458 (24.1)	21 (35.6)	437 (23.8)
5‐FU, *n* (%)			
Single agent	33 (1.7)	2 (3.4)	31 (1.9)
Combination therapy^a^	1211 (63.8)	28 (47.5)	1183 (64.3)

Count variables are presented as number (percentage). Continuous variables are expressed as median [25th percentile, 75th percentile].

5‐FU, 5‐flurouracil; FAC, fluoropyrimidine induced cardiotoxicity.

^a^ Patients on combination chemotherapy regimens including oxaliplatin, folinic acid, docetaxel, cetuximab, irinotecan, epirubicin, cisplatin, bevacizumab, mitomycin, and cyclophosphamide.

The top three most common underlying cancer diagnoses were colorectal (*n* = 922, 48.6%), breast (*n* = 414, 21.8%), and gastro‐oesophageal cancer (*n* = 275, 14.5%). Of the 5‐FU based regimens given, 5‐FU was delivered in both a bolus and continuous infusional form in 72% of cases, and in the remaining 28% of cases, it was delivered purely as a continuous infusion.

The baseline prevalence of cardiovascular risk factors is outlined in *Table* *2*. The median QRISK 3 score was 11.1% (4.2–21.6). The burden of smoking (40.6%), hypertension (37.9%), diabetes (20.9%), hypercholesterolaemia (23%), and CKD (9.6%) was high, with 23% of participants classed as obese.

**Table 2:** Comparison of baseline cardiovascular risk factors and pre‐existing cardiovascular disease in patients with and without fluoropyrimidine induced cardiotoxicity

Cardiac risk factors	Total cohort (*n* = 1898)	Patients with FAC (*n* = 59)	Patients without FAC (*n* = 1839)
QRISK3 score			
Median (IQR)	11.1 (4.2–21.6)	13.9 (5.1–27.3)	11 (4.2–21.4)
QRISK3 score category, *n* (%)			
Low risk (<10%)	897 (47.2)	24 (40.7)	873 (47.4)
Moderate risk (10.1–19.9%)	468 (24.7)	10 (16.9)	458 (24.9)
High risk (>20%)	533 (28.1)	25 (42.4)	508 (27.6)
Diabetes mellitus, *n* (%)	396 (20.9)	16 (27.1)	380 (20.7)
Hypercholesterolaemia, *n* (%)	436 (23.0)	15 (25.4)	421 (22.9)
Hypertension, *n* (%)	719 (37.9)	20 (33.9)	699 (38.0)
Smoking, *n* (%)			
Ex‐smoker	471 (24.8)	17 (28.8)	454 (24.7)
Current smoker	299 (15.8)	13 (22.0)	286 (15.6)
Family history of coronary disease, *n* (%)	100 (5.3)	11 (18.6)	89 (4.8)
Peripheral vascular disease, *n* (%)	18 (0.9)	1 (1.7)	17 (0.9)
CKD, *n* (%)	183 (9.6)	7 (11.9)	176 (9.6)
Obesity, *n* (%)	434 (22.9)	15 (25.4)	419 (22.8)
Pre‐existing cardiovascular disease, *n* (%)			
Known CAD	96 (5.1)	6 (10.2)	90 (4.9)
Previous medically managed MI	30 (1.6)	0 (0.0)	30 (1.6)
Previous PCI	53 (2.8)	5 (8.5)	48 (2.6)
Previous CABG	13 (0.7)	1 (1.7)	12 (0.7)
Valve disease	36 (1.9)	3 (5.1)	33 (1.8)
Non‐ischaemic cardiomyopathy	14 (0.7)	1 (1.7)	13 (0.7)
Clinical heart failure	28 (1.5)	2 (3.4)	26 (1.4)
Arrhythmias	101 (5.3)	5 (8.5)	96 (5.2)
Medications, *n* (%)			
Anticoagulation	234 (12.3)	9 (15.3)	225 (12.2)
ACEi/ARB	413 (21.8)	12 (20.3)	401 (21.8)
Beta‐blockers	195 (10.2)	12 (20.3)	183 (10.0)
Statins	522 (27.5)	19 (32.2)	503 (27.4)
Aspirin	156 (8.2)	8 (13.6)	148 (8.0)
Calcium channel blockers	383 (20.2)	9 (15.3)	374 (20.3)
Nitrates	47 (2.5)	3 (5.1)	44 (2.4)

ACE, angiotensin converting enzyme inhibitor; ARB, angiotensin receptor blocker; CABG, coronary artery bypass grafting; CAD, coronary artery disease; FAC, fluoropyrimidine induced cardiotoxicity; IQR, interquartile range; MI, myocardial infarction.

### Cardiovascular events during fluoropyrimidine chemotherapy

Among the study sample, 59 patients (3.1%) developed CV events, with most events (*n* = 23, 39.0%) occurring within the first cycle of treatment. The median onset time from initiation of chemotherapy administration to symptom onset was 5 days (IQR 2–7). New onset angina was the most common presentation (*n* = 38, 64.4% of events), followed by new onset AF (*n* = 8, 13.5%). ACS was observed in 7 patients (0.4% of total cohort), of whom *n* = 5 underwent PCI (*Table* *3*). Cardiovascular death or cardiac arrest occurred in three patients 0.2%. Of the 38 patients classified with new onset angina, all patients presented with typical anginal symptoms of chest/neck/jaw discomfort precipitated by exertion. Of these, three patients underwent invasive coronary angiography, four had CT coronary angiography, and 16 had a non‐invasive functional test (cardiovascular magnetic resonance) given that the chest pain had resolved on presentation to hospital.

**Table 3:** Clinical presentation of FAC and timing of event (*n* = 59)

Type of event	*N* (%)
Cardiovascular death	3 (5.1)
Acute coronary syndrome	7 (11.9)
Angina	38 (64.4)
Arrhythmia	8 (13.5)
Incident heart failure	3 (5.1)
Total	59

Time of first event occurrence	Median (IQR)
Time from last chemotherapy exposure (hours)	120 (48–168)
Time to event from time zero (first chemotherapy administration) (months)	3.8 (1.9–9.4)

The median age of those with CV events was 58 years (IQR 46–67); similar to those with no events (60 years, IQR 50–68). Thirty‐four of the 59 (57.6%) with events were male. Patients of South Asian ethnicity had the highest CV event rate (4.1%), compared with 3.9% in Caucasians and 1.7% in Black ethnicities. Event rates were higher with colorectal cancer (5.3%) than gastro‐oesophageal (2.2%) and breast cancer (0.5%).

Median QRISK3 score at baseline in those with CV events was 13.9% (IQR 5.1–27.3) compared with 11% (IQR 4.2–21.4) in those without events.

### Univariable associations

In univariable analysis, there was no association of age, sex, or ethnicity with incident CV events (*Table* *4*). There was an over two‐fold increase in risk in patients who were current smokers [HR 2.01, 95% CI 1.04–3.97), *P* = 0.03] compared with those who had never smoked. Every 1 unit increase in BMI was associated with a 5% increase in risk (HR 1.05, 95% CI 1.01–1.09, *P* = 0.01), with similar positive associations between BSA and FAC risk (HR 3.57, 95% CI 1.19–10.23, *P* = 0.02). A history of pre‐existing CAD was weakly associated with the development of FAC (HR 2.15, 95% CI 1.48–5.02, *P* = 0.04). There were no significant associations between CV events and other pre‐existing CV conditions (peripheral vascular disease, non‐ischaemic cardiomyopathy, arrhythmias, or valvular heart disease), or with many conventional cardiovascular risk factors such as hypertension, hypercholesterolaemia, diabetes, or CKD. The use of beta‐blockers was associated with CV events (HR 2.41, 95% CI 1.22–4.40, *P* = 0.01); no significant association was observed with any other medication class. Of the patients with cardiovascular events during treatment, *n* = 12 (20.3%) were taking beta‐blockers at baseline. Of these patients, *n* = 6 had hypertension, 2 had pre‐existing coronary artery disease, 2 patients were taking BB for paroxysmal atrial fibrillation, and 1 patient for longstanding palpitations, and the indication was unclear in the final patient.

**Table 4:** Univariable and multivariable association of covariates with fluoropyrimidine induced cardiotoxicity estimated using Cox‐proportional hazards models

	Model 1 (Univariable)	Model 2 (Multivariable)
Covariates	HR (95% CI), *P*‐value	Adj. HR (95% CI), *P*‐value
QRISK3 score		Not included
Low risk (<10%)	Reference level	
Moderate risk (10.1–19.9%)	0.73 (0.33–1.49), 0.44	
High risk (>20%)	2.25 (1.11–4.93), 0.03	
Age	1.00 (0.97–1.01), 0.64	0.98 (0.96–1.01), 0.13
Sex		
Female	Reference level	Reference level
Male	1.46 (0.86–2.50), 0.16	1.40 (0.41–1.24), 0.24
Ethnicity		Not included
White	Reference level	
Black	0.49 (0.14–1.23), 0.18	
South Asian	1.21 (0.52–2.52), 0.63	
Other Asian	NA	
Not stated	NA	
Use of beta‐blockers	2.41 (1.22–4.40), 0.01	2.33 (1.04–5.19), 0.04
Smoking history		1.50 (0.88–2.57), 0.14
Current smoker	2.01 (1.04–3.97), 0.03	
Ex‐smoker	1.45 (0.78–2.63), 0.23	
Never smoked	Reference level	
Body mass index	1.05 (1.01–1.09), 0.01	1.04 (1.00–1.08), 0.04
Body surface area	3.57 (1.19–10.23), 0.02	Not included
Chemotherapy type		Not included
Capecitabine	1.54, (0.91–2.59), 0.10	
5‐FU	Reference level	
Treatment intent		Not included
Curative	Reference level	
Palliative	0.52, 0.30–1.18, 0.06	
Pre‐existing CAD	2.15 (1.48–5.02), 0.04	1.13 (0.39–3.25), 0.83
Non‐ischaemic cardiomyopathy	5.11 (0.29–23.60), 0.11	Not included
Valve disease	2.01 (0.49–5.51), 0.24	Not included
Peripheral vascular disease	1.50 (0.08–6.82), 0.69	Not included
Hypertension	0.80 (0.46–1.35), 0.41	Not included
Hypercholesterolaemia	1.26 (0.68–2.21), 0.45	Not included
Diabetes mellitus	1.29 (0.70–2.24), 0.39	Not included
Arrhythmia	1.96 (0.68–4.46), 0.15	Not included
Chronic kidney disease	1.17 (0.48–2.41), 0.70	Not included

Results are the hazard ratio (HR), 95% confidence interval (CI), and *P*‐value representing association of each covariate with FAC. Model 1 represents univariate associations with each covariate inserted separately into the model. Covariates with significant univariable associations in Model 1, where taken forward to Model 2, which represents multivariable association of all stated covariates entered simultaneously into the model. Each covariate excluded from the multivariable model was marked with ‘not included’ in the model 2 column.

There were no differences in frequency of events between patients treated with curative or palliative intent. DPD assessment was performed in 468 (24.7%) patients. Among these, 36/468 (7.7%) were heterozygous for DPD mutation; none were homozygous. Patients with mutations received dose modifications to their prescribed fluoropyrimidine chemotherapy. Events were no more common in patients with DPD heterozygous mutations (HR 2.29, 95% CI 0.67–5.97, *P* = 0.13).

### Multivariable associations

In multivariable models, higher BMI remained significantly associated with the development of FAC (HR 1.04, 95% CI 1.00–1.08, *P* = 0.04) as did the use of beta‐blockers at baseline (HR 2.33, 95% CI 1.04–5.19, *P* = 0.04). The associations of smoking history and pre‐existing CAD with FAC were attenuated in multivariable models.

### QRISK3

**Figure 1:**
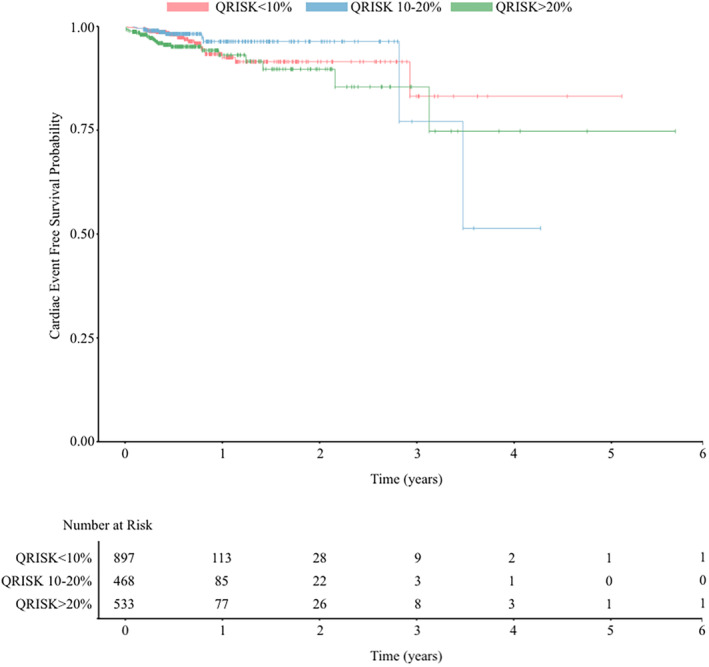
Kaplan–Meier survival curve comparing CV event‐free survival between patients in different QRISK3 groups. The *P* value was calculated using the log‐rank test.

Of the *n* = 59 patients who developed CV events, 25 (42.4%) had a QRISK3 score of >20% compared with 10/59 (16.9%) with moderate and 24/59 (40.7%) with low risk QRISK 3 scores (*Figure* *1*). In univariable analysis, QRISK3 score >20% was significantly associated with incident FAC (HR 2.25, 95% CI 1.11–4.93, *P* = 0.03).

### Outcomes in patients with fluoropyrimidine‐associated cardiotoxicity events

In those who experienced FAC, 44.1% (*n* = 26) had their fluoropyrimidine chemotherapy discontinued because of the event (three had died from sudden cardiac death). Of these patients, 46% (*n* = 12) were switched to alternative chemotherapy regimens. In one patient (1.7%), the event occurred in the final cycle of treatment, and no further treatment had been planned. From the remaining 24 patients, 3 (12.5%) had a recurrence of troponin negative cardiac‐sounding chest pain.

Thirty‐two patients (54.2%) were initiated on cardioprotective medications (oral nitrate and CCBs where appropriate) and rechallenged with fluoropyrimidine chemotherapy, with symptom recurrence (troponin‐negative anginal‐type pain) reported in a minority (*n* = 2, 6.3%). Of these patients rechallenged, initial cardiac presentations had been with new onset angina (*n* = 23 patients), ACS (*n* = 1), atrial fibrillation (*n* = 6), and heart failure (*n* = 2).

### Overall survival

**Figure 2:**
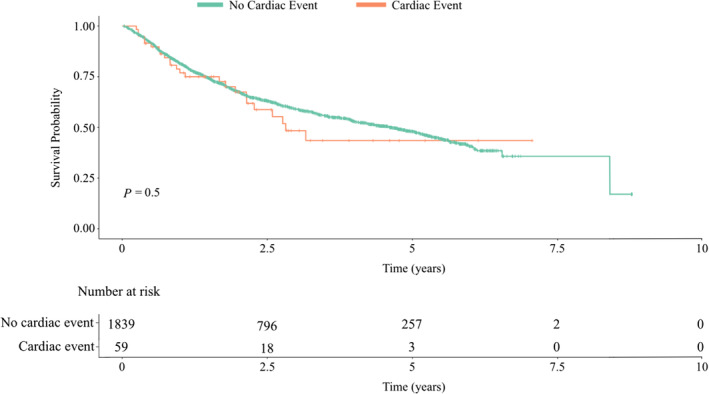
Kaplan–Meier survival curve comparing overall survival between patients who developed CV events. The *P* value was calculated using the log‐rank test.

Across all patients, there was no significant difference in overall survival between patients who developed FAC and patients who did not, HR 1.15 (CI 0.92–1.49), *P* = 0.50 (*Figure* 2). Of patients with events, there was no significant difference in overall survival between patients who were rechallenged and those who were not, *P* = 0.1.

## Discussion

In this large retrospective observational study from a single UK tertiary centre, we found the incidence of CV events during fluoropyrimidine chemotherapy to be 3.1%. The patients included represent an unselected multi‐ethnic, multi‐morbid clinical cohort across a range of malignancies, receiving neoadjuvant and adjuvant curative, and palliative treatment. New onset angina (64.4%) was the most common FAC presentation. Cardiac arrest and cardiovascular death were rare, occurring in 0.2% of our entire cohort, despite high rates of pre‐existing CVD and risk factors. To our knowledge, this is the first study to have used a conventional cardiovascular risk assessment tool (QRISK3 risk calculator) to provide an objective baseline cardiovascular assessment and assess the relationship with FAC. Patients with the highest QRISK3 scores (>20% 10‐year risk of heart attack or stroke) had significantly greater risk of FAC, compared with those in the lowest risk category. We also found that an increased BMI and the use of beta‐blockers at baseline had a significant association with FAC on multivariable analysis. A history of pre‐existing coronary artery disease (5.1% of patients across the cohort) was not associated with the development of FAC on multivariable analysis. There was no significant difference in overall survival between patients who developed FAC and those who did not. Recurrent events were uncommon in patients re‐challenged with fluoropyrimidine following FAC.

Although the quoted incidence of FAC varies greatly across studies, our findings of 3.1% are comparable to other studies from across Europe and the United States.^11^, ^13^, ^16^, ^17^ The most common manifestation of FAC was incident angina and ACS, which is also in keeping with other series.^11^, ^13^, ^16^ Rates of AF across the entire cohort were low at 0.4%, consistent with other studies reporting 1.1% and 0.4%.^16^, ^18^

Improving risk prediction in patients undergoing fluoropyrimidine chemotherapy is of high clinical importance. The ICOS ESC‐HFA position statement did not include risk prediction recommendations for fluoropyrimidines owing to insufficient evidence.^14^ Guidance is similarly limited in the recent ESC cardio‐oncology guidelines^6^ regarding baseline assessment and monitoring during fluoropyrimidine chemotherapy. Although our data found that patients in the highest risk group (QRISK3 > 20%) are at increased risk of acute cardiotoxicity, those with QRISK 3 10–20% were not at increased risk when compared to those with QRISK 3 < 10% and cardiovascular events occurred across all risk groups, highlighting the need for improved specific risk prediction instruments for use in this setting.

Previous studies investigating the relationship between baseline cardiovascular risk factors and pre‐existing cardiovascular disease with the development of FAC have produced conflicting results. These studies vary in their designs, cohort size, sample demographics, and the nature of cardiac outcomes assessed. A retrospective study of 452 women with breast cancer treated with capecitabine, reported higher risk of CV events in patients with cardiac comorbidities (including ischaemic heart disease, significant history of arrhythmia, and LV dysfunction),^16^ smoking, and hyperlipidaemia; however, these results may not be generalizable to male patients. In another retrospective review of 668 patients treated with fluoropyrimidines, both pre‐existing cardiac disease and renal disease were significantly associated with CV events.^11^ In contrast, other studies have reported no significant relationship between pre‐existing CVDs and risk factors and higher risk of events.^19^, ^20^ A recent meta‐analysis,^21^ including 22 studies and 21 000 patients, found that patients with pre‐existing CVD had significantly higher risks of events (pooled RR = 3.26, 95% CI = 2.15–4.95, 95% PI = 1.02–10.43, *I* ^2^ = 46%). There was, however, significant heterogeneity between the 13 studies specifically assessing this association, with diverse patient populations, cohort sizes, and study designs. In a subsequent retrospective review of 4019 patients treated with fluoropyrimidines, the incidence of coronary artery vasospasm was 2.1%.^17^ No other clinical presentations of FAC were considered, and patients at increased risk were found to be younger and less likely to have traditional cardiovascular risk factors compared with those who did not develop coronary artery vasospasm.

Fluoropyrimidine dosing is calculated based on BSA; hence, larger patients receive higher total chemotherapy doses. We show that higher BSA was positively associated with the development of FAC, in line with a previous study where patients with a higher BSA were more likely to develop early, severe systemic toxicity (not limited to cardiac toxicity). There are however no prior studies which have assessed the relationship of FAC with BSA. Two prior studies have investigated the relationship of BMI with FAC, with conflicting results. A retrospective analysis of 916 colorectal patients treated with fluoropyrimidines found that a BMI > 22.97 kg/m^2^ was significantly associated with FAC but only in those treated with 5‐FU, not capecitabine.^22^ In contrast, a pooled analysis of five randomized controlled trials of colorectal patients treated with fluoropyrimidines did not find BMI to be associated with FAC,^23^ likely due to differences between clinical trial, and real‐world, patient groups. The differential associations of BMI and BSA with cardiotoxicity events highlight the potential for different risk susceptibility dictated by body composition profiles.^24^, ^25^ This would indeed be congruent with a growing body of research highlighting the importance of body composition in driving adverse health risks. More detailed profiling of body composition for greater precision of cardiotoxicity estimates requires further evaluation.

The association of DPD deficiency and FAC has not been previously assessed. Only a quarter of our cohort had DPD genetic testing, which was mandated in our centre from 2018. Within this limitation, we did not see a significant association of DPD deficiency and the development of FAC. In line with local protocols, patients with DPD genetic mutations were administered reduced doses of fluoropyrimidine chemotherapy, which may explain this lack of association with FAC. These results, and the association of BSA with FAC, suggest that the total dose of fluoropyrimidines may influence risk of FAC.

We showed that the use of beta‐blockers at baseline was significantly associated with the development of FAC. The most commonly postulated mechanism of FAC is coronary artery vasospasm which may be via endothelial dependent or independent mechanisms.^26^, ^27^, ^28^ Beta‐blockers are generally not recommended in the treatment of vasospastic angina as they can aggravate alpha‐mediated vasoconstriction, and while our study is not designed to assess the pathophysiology of FAC, this may be the underlying mechanism. It is however possible that the association of FAC with beta‐blocker usage may be surrogate for underlying established cardiovascular disease, despite our finding of a continued association after adjustment in multi‐variable analysis. This relationship has not been found in other studies, and in contrast, Zafar et al. found that fluoropyrimidine‐associated vasospasm were less likely to be in patients receiving beta‐blockers.^17^

In patients who develop FAC, the decision to rechallenge is controversial due to balancing concerns regarding symptom recurrence and risk of sudden cardiac death with the scarcity of evidence for efficacy of alternative cancer therapies. In one of the largest studies to date, assessing safety of rechallenge, of 115 patients developing coronary artery vasospasm on fluoropyrimidine treatment, 81 patients were rechallenged. The majority of these received nitrates or CCB.^29^ Symptoms recurred in 19% of those who received cardioprotective medications, although no MI events were recorded. The authors concluded that fluoropyrimidine rechallenge combined with CCBs or nitrates under guidance from a cardio‐oncology service is safe. In another case series of 11 patients who had experienced FAC, there was 100% success of rechallenge using a combined strategy of switching to bolus 5‐FU from continuous infusional and the addition of vasodilator cardioprotective medications.^30^ In our study, 54% of patients were rechallenged and of those, there was a recurrence of symptoms in only 6.3%, with no cardiovascular deaths. This suggests that rechallenge can be safely attempted with close monitoring.

## Study limitations

The main limitation with this study was the retrospective design and the small number of FAC events, which necessitated the use of a composite outcome. It is possible that the individual cardiotoxicities have different predictors; however, we cannot derive disease‐specific associations due to low power. The true incidence of FAC may be underestimated due to missing or incomplete medical record data. Multicentre analyses are required to assess generalizability of our findings. A limitation of our retrospective data collection was missingness in the ethnicity category. This limits our ability to detect ethnicity differential relationships. Prospective analyses with standardized data collection of demographic variables or use of databases where this information is more completely recorded would be useful. A prospective observational cohort study in a multi‐ethnic population is currently underway (NCT05159479). It is also likely that patients at highest CV risk did not receive fluoropyrimidines due to cancer physician concerns related to FAC; hence, studies addressing this evidence gap are needed. This study considers the value of QRISK3 in detecting cardiotoxicity events for 5‐FU chemotherapy. A range of other CVD risk scores are available, and evaluation of which of these is most useful in the context of cardiotoxicity warrants further investigation in dedicated future studies. The study was a retrospective study including consecutive patients starting fluoropyrimidine therapy from 2014 to 2020, predating publication of the ESC Cardio‐Oncology guidelines. Hence, most patients did not have formal CV risk stratification performed by their oncologists prior to treatment, and biomarkers were not available in the majority. Similarly, due to the retrospective nature of this study, we did not feel it was possible to reliably determine whether presentations of angina were specifically related to coronary artery spasm due to a lack of available ECG documentation in all patients at the time of symptoms to further support the diagnosis, and therefore, we classified our patients with typical angina symptoms in a singular category. While the association between beta‐blockers and cardiotoxicity remained significant incorporating pre‐existing CAD in the model, it is possible some of the remaining patients may also have had CAD, which had not been ascertained from the history. Finally, while overall survival in oncology depends on type and stage of cancer, we could not explore this in our analysis due to our limited sample size due to limited statistical power. We have presented the breakdown of patient characteristics in those with FAC (*Table* *S2*).

## Conclusions

Overall cardiovascular event rates are low in patients receiving fluoropyrimidine chemotherapy but are more common in patients living with obesity and in those taking beta‐blockers at baseline. The use of the QRISK3 calculator may be of value at baseline prior to fluoropyrimidine chemotherapy to identify those at highest risk of CV events; however, specific risk calculators are needed for improved discrimination.

## Conflict of interest

CM is a CoFounder of MyCardiumAI. SS has received consultancy fees from Pfizer Cachexia board and EISAI.

## Funding

AA is funded by British Heart foundation Clinical Research Training Fellowship (FS/CRTF/21/24134). ZRE recognizes the National Institute for Health and Care Research (NIHR) Integrated Academic Training Programme that supported her Academic Clinical Lectureship post. CM is supported directly and indirectly from the NIHR Biomedical Research Centres at University College London Hospitals and Barts Health NHS Trusts.

## Supporting information


**Table S1.** Supporting Information.
**Table S2.** Breakdown of characteristics of patients with FAC.
