# Supplementary material for: Predicting cardiovascular events with fluoropyrimidine chemotherapy using a standard cardiovascular risk calculator
Source: ESC Heart Fail. 2024 Jun 6;11(5):3041–51. doi: 10.1002/ehf2.14879 (PMC11424348; doi:10.1002/ehf2.14879)
Supplement: Supplementary file 1 — Table S1. Supporting Information. Table S2. Breakdown of characteristics of patients with FAC. [file EHF2-11-3041-s001.docx]

**Supplementary Table 1**

|  | **ICD-10 classifications** |
| --- | --- |
| Chest pain, unspecified | R07 |
| Angina pectoris with documented spasm | I20.1 |
| Myocardial infarction | I21 |
| Non ST elevation MI (NSTEMI) | I21. 4 |
| Acute coronary syndrome (ACS) | I24.9 |

**Supplementary Table 2- Breakdown of characteristics of patients with FAC.**

| **Characteristic** | **Curative intent, n=31 (52.5%)** | **Palliative intent, n=28 (47.5%)** |  |
| --- | --- | --- | --- |
| **Age at diagnosis (years)**  **Median (IQR)** | **50 (42-65)** | **62 (54-68)** |  |
|  |  |  |  |
| **Sex** |  |  |  |
| **Female** | **15 (48.4)** | **10 (35.7)** |  |
| **Male** | **16 (51.6)** | **18 (64.3)** |  |
| **Ethnicity** |  |  |  |
| **White** | **15 (48.4)** | **16 (57.1)** |  |
| **Black** | **2 (6.5)** | **2 (7.1)** |  |
| **South Asian** | **6 (19.4)** | **2 (7.1)** |  |
| **Other Asian** | **0 (0.0)** | **0 (0.0)** |  |
| **Not stated** | **8 (25.8)** | **8 (28.6)** |  |
| **Performance Status**  **0**  **1**  **2**  **3**  **4** | **14 (45.2)**  **16 (51.6)**  **1 (3.2)**  **0 (0.0)**  **0 (0.0)** | **14 (50.0)**  **12 (42.9)**  **2 (7.1)**  **0 (0.0)**  **0 (0.0)** |  |
| **Primary site** |  |  |  |
| **Colorectal** | **28 (90.3)** | **18 (64.3)** |  |
| **Breast** | **1 (3.2)** | **1 (3.6)** |  |
| **Oesophageal** | **1 (3.2)** | **2 (7.1)** |  |
| **Pancreas** | **0 (0.0)** | **2 (7.1)** |  |
| **Stomach** | **1 (3.2)** | **2 (7.1)** |  |
| **Head and neck** | **0 (0.0)** | **0 (0.0)** |  |
| **Hepatobiliary** | **0 (0.0)** | **2 (7.1)** |  |
| **Bladder** | **0 (0.0)** | **0 (0.0)** |  |
| **Cancer of unknown primary (CUP)/Malignancy of unknown origin (MUO)** | **0 (0.0)** | **0 (0.0)** |  |
| **Other** | **0 (0.0)** | **1 (3.6)** |  |
| **DPD status** |  |  |  |
| **Negative** | **15 (48.4)** | **8 (28.6)** |  |
| **Heterozygous** | **3 (9.7)** | **1 (5.6)** |  |
| **Homozygous** | **0 (0.0)** | **0 (0.0)** |  |
| **Not tested** | **13 (41.9)** | **19 (67.9)** |  |
| **Chemotherapy** |  |  |  |
| **Capecitabine**  **5FU** | **20 (64.5)**  **11 (35.5)** | **9 (32.1)**  **19 (67.9)** |  |
